# Management of Patients Receiving Anticoagulation Therapy in Dental Practice: A Systematic Review

**DOI:** 10.3390/healthcare12151537

**Published:** 2024-08-02

**Authors:** Francesco Inchingolo, Angelo Michele Inchingolo, Fabio Piras, Laura Ferrante, Antonio Mancini, Andrea Palermo, Alessio Danilo Inchingolo, Gianna Dipalma

**Affiliations:** 1 Department of Interdisciplinary Medicine, University of Bari “Aldo Moro”, 70124 Bari, Italy or a.inchingolo3@studenti.uniba.it (A.M.I.); or laura.ferrante@uniba.it (L.F.); or antonio.mancini@uniba.it (A.M.); or a.inchingolo1@studenti.uniba.it (A.D.I.); or gianna.dipalma@uniba.it (G.D.); 2 College of Medicine and Dentistry, Birmingham B4 6BN, UK; andrea.palermo2004@libero.it

**Keywords:** anticoagulant, dentistry, tranexamic acid, warfarin, vitamin K antagonists, NAO, TAO

## Abstract

Background: Anticoagulant drugs are a valuable tool for minimizing thrombotic risks in at-risk patients. The purpose of this study is to conduct a literature review highlighting the management of these drugs during daily clinical dental practice. Materials and Methods: We limited our search to English-language papers published between 1 January 1989, and 7 March 2024, in PubMed, Scopus and Web of Science that were relevant to our topic. In the search approach, the Boolean keywords “anticoagulant AND dentistry” were used. Results: Twenty-five clinical trials were included for final review from 623 articles obtained from the databases Web of Science (83), PubMed (382), and Scopus (158), eliminating duplicates and 79 off-topic items, resulting in 419 articles after removing 315 entries and confirming eligibility. Overall, these studies support the use of local hemostatic measures to manage the risk of bleeding in patients on anticoagulant therapy undergoing dental procedures and highlight the importance of greater education and collaboration among healthcare professionals. Conclusions: Research and clinical investigation have improved understanding and management of dental procedures in patients undergoing anticoagulant or antiplatelet therapy. Hemostatic agents, clinical protocols, risk factors, and continuous education are essential for navigating the complexities of anticoagulant therapy, ensuring optimal outcomes and enhancing patient well-being.

## 1. Introduction

**Figure 1:**
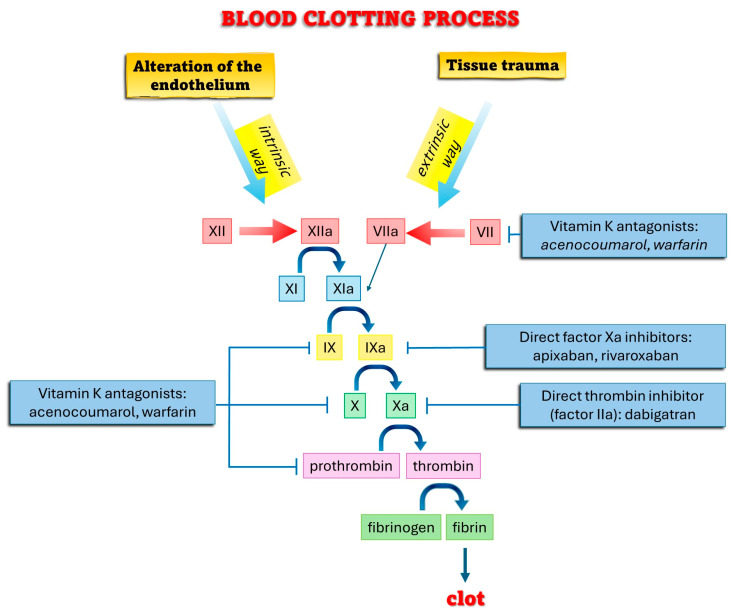
Blood clotting process.

Anticoagulants play a vital role in the management of various thromboembolic disorders, exerting their effects through different mechanisms and pharmacokinetic profiles. Understanding the pharmacological properties of these agents is essential for tailoring perioperative management strategies, particularly in dental practice. This systematic review aims to address the specific problem of managing bleeding risks in patients undergoing dental procedures while on anticoagulant therapy [1]. Anticoagulants are essential in the prevention and treatment of thromboembolic disorders, and they include a variety of agents such as vitamin K antagonists (VKAs), direct oral anticoagulants (DOACs), and low-molecular-weight heparins (LMWHs). Each of these classes has distinct mechanisms of action, pharmacokinetic properties, and implications for clinical use, especially in the context of dental procedures [2,3]. Vitamin K antagonists (VKAs), such as warfarin, have been mainstays of anticoagulant therapy for decades. These agents inhibit the synthesis of vitamin K-dependent clotting factors in the liver, primarily factors II, VII, IX, and X (Figure 1) [4].

VKAs, such as warfarin, have been the cornerstone of anticoagulant therapy for many years. They inhibit the synthesis of vitamin K-dependent clotting factors in the liver, primarily factors II, VII, IX, and X. Warfarin’s mechanism of action involves interfering with the gamma-carboxylation of glutamic acid residues on these factors, thereby impairing their functional activity [5,6,7,8,9,10,11,12,13]. Despite their efficacy in preventing thromboembolic events, VKAs have several limitations. These include a slow onset of action, a narrow therapeutic window, and a variable response influenced by genetic and environmental factors [14]. Regular monitoring of the international normalized ratio (INR) is required to maintain therapeutic efficacy while minimizing the risk of bleeding. Additionally, VKAs are susceptible to numerous drug–drug and drug–food interactions, necessitating careful dose adjustments and close monitoring [15].

Direct oral anticoagulants (DOACs), which include direct thrombin inhibitors (e.g., dabigatran) and direct factor Xa inhibitors (e.g., apixaban, rivaroxaban, edoxaban), represent a newer class of anticoagulants that have emerged as preferred alternatives to VKAs in many clinical scenarios. DOACs target specific coagulation factors, providing more predictable pharmacokinetics, rapid onset of action, and fixed dosing regimens without the need for routine monitoring. These attributes, along with a superior safety profile and fewer drug interactions, make DOACs particularly attractive for patients requiring anticoagulation therapy [15].

Low-molecular-weight heparins (LMWHs), such as enoxaparin and dalteparin, are injectable anticoagulants commonly used in both inpatient and outpatient settings. These agents inhibit factor Xa and, to a lesser extent, thrombin, resulting in anticoagulant effects. LMWHs have predictable pharmacokinetics, allowing for weight-based dosing without the need for regular monitoring. They are particularly useful in situations where oral anticoagulation is contraindicated or impractical, such as during pregnancy, in patients with acute venous thromboembolism, or for perioperative bridging in patients with mechanical heart valves. Despite the differences in their mechanisms of action and pharmacokinetic profiles, all anticoagulants carry an inherent risk of bleeding complications. Dental practitioners must understand these pharmacological properties to make informed decisions about perioperative management and minimize the risk of adverse events [16].

The safe management of patients receiving anticoagulation therapy undergoing dental procedures requires a systematic approach to risk stratification. Dental procedures vary widely in terms of invasiveness, duration, and associated bleeding risk. Therefore, individualized assessment and management strategies are crucial for each patient [17].

Low-risk dental procedures, such as routine dental cleanings, simple restorations, and non-surgical extractions of non-impacted teeth, typically pose minimal bleeding risk and can often be safely performed without interrupting anticoagulation therapy [18]. These procedures involve minimal manipulation of soft tissues and are unlikely to cause significant bleeding complications in patients receiving anticoagulants. However, dental practitioners should still exercise caution and employ appropriate hemostatic measures, such as the use of local hemostatic agents, to minimize bleeding risk and ensure patient safety [19].

Moderate-risk dental procedures, including surgical extractions, multiple tooth extractions, and periodontal surgeries, carry a higher risk of bleeding complications and may warrant temporary interruption of anticoagulation therapy [20,21,22,23,24]. The decision to continue or interrupt anticoagulation therapy depends on a comprehensive assessment of the patient’s thrombotic and bleeding risks. This assessment should consider the indication for anticoagulation, the patient’s underlying medical conditions, and the complexity of the dental procedure. In some cases, alternative strategies, such as bridging therapy with LMWHs, may be employed to balance the risks of thrombosis and bleeding [25].

High-risk dental procedures, such as extensive oral surgeries, implant placements, and major bone grafting procedures, pose significant bleeding risk and often require temporary cessation of anticoagulation therapy. In these cases, dental practitioners must collaborate closely with the patient’s healthcare providers to develop a comprehensive perioperative management plan [26,27]. This plan should aim to minimize the risk of postoperative bleeding, hematoma formation, and thromboembolic events. Effective communication and coordination between the dental team and other healthcare professionals are essential to optimize patient safety and clinical outcomes [28,29].

To minimize bleeding complications during dental procedures, a multifaceted approach involving local hemostatic measures, pharmacological interventions, and interdisciplinary collaboration is essential [30].

Direct pressure with sterile gauze is the initial step in achieving hemostasis during dental procedures. Suturing to close wounds and approximate tissue edges can also be effective. Additionally, the use of topical hemostatic agents, such as oxidized cellulose, gelatin sponges, and fibrin sealants, can enhance hemostasis [31,32,33,34,35]. These agents work by promoting clot formation and stabilizing the clot at the site of bleeding. Pharmacological interventions, such as tranexamic acid and ε-aminocaproic acid, can be employed to minimize bleeding risk in patients receiving anticoagulation therapy. Tranexamic acid mouth rinse, in particular, has been shown to effectively reduce bleeding in dental procedures. It is a cost-effective and well-tolerated option, making it a valuable adjunct in managing bleeding risk in anticoagulated patients [36,37,38,39,40,41,42]. The use of these pharmacological agents should be guided by evidence-based protocols and tailored to the individual patient’s needs. The perioperative management of patients receiving anticoagulation therapy in dental practice necessitates a comprehensive approach that involves close collaboration with other healthcare providers. This includes cardiologists, hematologists, and primary care physicians, who can provide valuable insights into the patient’s overall medical condition and the risks associated with anticoagulation therapy. By leveraging the international literature, evidence-based guidelines, and expert recommendations, dental practitioners can make informed decisions regarding anticoagulant management, ensuring optimal patient outcomes and enhancing the quality of care provided [43].

The primary objective of this systematic review is to evaluate the existing evidence on bleeding risks associated with different anticoagulants during dental procedures. By synthesizing data from various studies, this review aims to provide evidence-based recommendations for clinical practice [44]. The review seeks to identify the best practices for managing anticoagulated patients in dental settings, focusing on minimizing bleeding complications while ensuring effective thromboembolic protection. Additionally, the review aims to highlight gaps in the current literature and suggest areas for future research to further optimize the management of anticoagulated patients undergoing dental procedures. Anticoagulants are critical in preventing and treating thromboembolic disorders, but their use complicates the management of dental procedures due to the inherent risk of bleeding [45]. Understanding the pharmacological properties of VKAs, DOACs, and LMWHs, along with appropriate risk stratification of dental procedures and effective bleeding management strategies, is essential for dental practitioners [46]. This systematic review aims to provide a comprehensive overview of the current evidence and offer practical guidelines to enhance patient safety and clinical outcomes in this complex clinical scenario. Through ongoing research and interdisciplinary collaboration, dental professionals can navigate the challenges of managing anticoagulation therapy in the perioperative setting, ultimately improving patient care and satisfaction [47].

## 2. Materials and Methods

### 2.1. Protocol and Registration

This review was carried out in accordance with PRISMA (Preferred Reporting Items for Systematic Reviews and Meta-Analyses) guidelines, and it was registered under the number CRD530556 on PROSPERO (The International Prospective Register of Systematic Reviews) [48].

### 2.2. Search Processing

We limited our search to English-language papers published between 1 January 1989, and 7 March 2024, in PubMed, Scopus and Web of Science that were relevant to our topic. In the search approach, the Boolean keywords “anticoagulant” AND “dentistry” and “anticoagulant” AND “oral surgery” were used. We selected these phrases because they most accurately reflected our investigation’s aim, which was to gain additional insight into the interaction between anticoagulant drugs and dentistry therapy (Table 1).

### 2.3. Inclusion Criteria

Three reviewers evaluated all relevant papers based on the following chosen criteria: (1) solely human subjects studies, (2) complete text and (3) scientific studies evaluating the management of anticoagulant drugs during clinical dental practices, in particular oral surgery treatments. The following process was used to construct the PICO model:

- Criteria: Application in the present study;
- Population: Human subjects with cardiac and coagulation pathologies;
- Intervention: Oral surgery;
- Comparison: Control group; antiplatelet group; heparin group; local hemostatic agents;
- Outcome: Evaluation of anticoagulant drugs during dentistry treatment;
- Study design: Clinical trials.

### 2.4. Exclusion Criteria

Articles written in languages other than English, ineligible study designs, ineligible outcome measures, ineligible populations, case studies, reviews, and animal studies were among the exclusion criteria.

### 2.5. Data Processing

Author disagreements on the choice of articles were addressed and settled.

### 2.6. Article Identification Procedure

The appropriateness evaluation was performed independently by two reviewers, F.I. and F.P. An additional manual search was conducted to increase the number of articles available for full-text analysis. English-language articles that met the inclusion criteria were taken into consideration, and duplicates and items that did not qualify were marked with the reason they were not included.

### 2.7. Study Evaluation

The article data were independently evaluated by the reviewers using a special electronic form designed according to the following categories: authors, year of study, aim of the study, materials and methods, and results. The special electronic form used is ROBINSON. This tool was developed to provide a systematic and comprehensive assessment of the risk of bias in studies that do not use randomization to assign participants to intervention groups.

### 2.8. Quality Assessment

Two reviewers, F.P. and L.F., evaluated the included papers’ quality using the ROBINS-I tool.

ROBINS-I was created to evaluate the potential for bias in the outcomes of non-randomized trials comparing the health impacts of two or more therapies. Each of the seven evaluated points was given a bias degree: confounding, participant selection, intervention classification, deviations from intended interventions, missing data, outcome measurement, and choice of reported results. Reviewers were instructed on how to use the ROBINS-I tool and adhered to its guidelines. In cases of disagreement, F.I., the third reviewer, was consulted until a consensus was reached. This process ensured objectivity and uniformity in the assessments. The use of ROBINS-I allowed for an extensive assessment of potential biases in the non-randomized studies included in this review. It contributed to the overall evaluation of the quality and reliability of the results by highlighting the strengths and weaknesses of the evidence base. By considering the risk of bias, the reviewers were able to reach more informed interpretations and conclusions based on the available data.

## 3. Results

A total of 623 papers were obtained from the databases Web of Science (83), PubMed (382), and Scopus (158).

**Figure 2:**
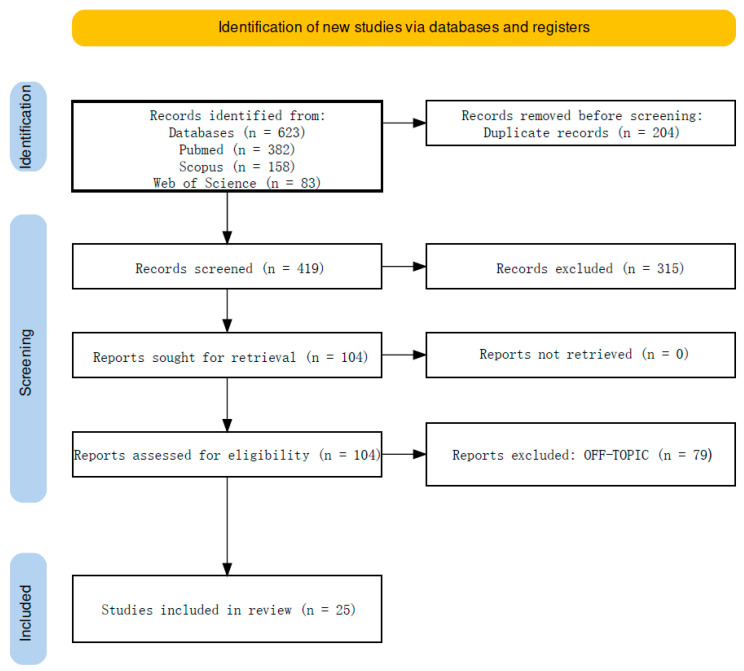
PRISMA flowchart of the literature search and article inclusion process.

After eliminating duplicates (204), 419 articles remained. Screening of titles and abstracts led to the exclusion of 315 entries (14 were in animals, 213 were reviews, 88 were off-topic). The remaining 104 papers were obtained and assessed for eligibility, resulting in the elimination of 79 items due to being off-topic. The qualitative analysis of the final 25 articles is included in this study, and the findings of each study are summarized in Figure 2.

The summary table (Table 2) details the authors, year of study, aim, materials used, and key results. These studies cover various aspects of dental procedures in patients on anticoagulant or antiplatelet therapy.

### Quality Assessment and Risk of Bias of Included Articles

**Figure 3:**
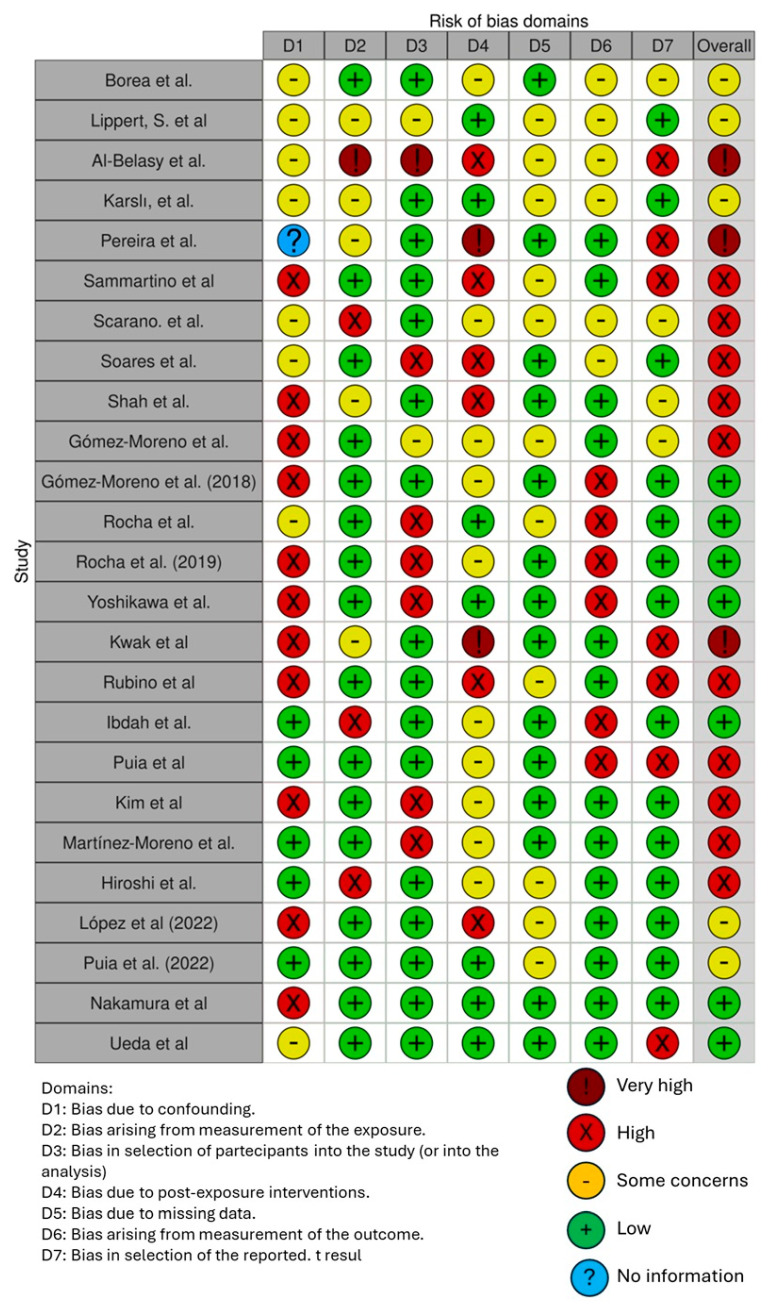
Bias assessment [3,8,47,49,50,51,52,53,54,55,56,57,58,59,60,61,62,63,64,65,66,67,68,69,70].

The risk of bias in the included studies is reported in Figure 3. Regarding the bias due to confounding, most studies have a high risk. The bias arising from measurement is a parameter with low risk of bias. Many studies have low risk of bias due to bias in selection of participants. Bias due to post exposure cannot be calculated due to high heterogeneity. The bias due to missing data is low in many studies. Bias arising from measurement of the outcome is low. Bias in the selection of the reported results is high in most studies. The final results show that 7 studies have low risk of bias, 2 have a very high risk of bias, and eleven have high risk of bias.

## 4. Discussion

Al-Belasy et al.’s study explores Histoacryl glue’s hemostatic efficacy in oral surgery patients on warfarin, addressing bleeding risk management challenges and traditional approaches to prevent complications [3,71,72,73,74,75,76]. The study evaluates the effectiveness of anticoagulation therapy during oral surgery, revealing immediate hemostasis with Histoacryl glue compared to delayed hemostasis in control groups [77,78,79,80,81]. The study also identifies a significant reduction in postoperative bleeding complications in patients treated with Histoacryl glue, highlighting its effectiveness as a local hemostatic agent in warfarin-treated patients undergoing oral surgery [82,83,84,85,86]. The study emphasizes the need for alternative anticoagulation management and the feasibility of oral surgery in patients on anticoagulant therapy, but acknowledges limitations like small sample size and suggests future research on standardized protocols [65,87,88,89,90]. Borea et al.’s study explores tranexamic acid mouthwash’s efficacy as a hemostatic agent in oral surgery patients receiving anticoagulant therapy, suggesting local antifibrinolytic therapy may prevent bleeding complications post-surgery [8,91,92,93,94,95]. Thirty patients with cardiac valve prostheses and receiving oral anticoagulants were enrolled in the study. Patients were divided into two groups: one receiving tranexamic acid mouthwash without discontinuing anticoagulant therapy and the other receiving a placebo mouthwash with anticoagulant therapy discontinuation [96,97,98]. The mouthwash regimen involved rinsing four times daily for seven days post-surgery. Results showed no significant difference in bleeding incidence between the tranexamic acid and control groups. Despite reduced coagulation levels during surgery, tranexamic acid demonstrated efficacy in controlling bleeding [99,100,101,102,103]. The study suggests that local antifibrinolytic therapy with tranexamic acid mouthwash could effectively prevent bleeding after oral surgery in patients receiving anticoagulant therapy, eliminating the need to discontinue anticoagulant therapy before surgery and avoiding thromboembolism risks. Further research is needed to validate these findings and explore the broader implications of local antifibrinolytic therapy in oral surgery for anticoagulant-treated patients [69,104,105,106,107]. The study of Hiroshi et al. explores the frequency of hemorrhage following tooth extraction in patients undergoing treatment with DOACs. This research represents a significant advancement in our understanding of the safety profile associated with DOAC therapy in dental procedures. First and foremost, let us acknowledge the meticulous nature of this study [66,108,109,110,111,112,113]. A study in Japan found that patients treated with dabigatran or rivaroxaban experienced similar post-extraction hemorrhages to those on traditional warfarin treatment, challenging existing paradigms and prompting a reconsideration of anticoagulant therapy in dental settings [114,115,116]. The study found that patients on dabigatran experienced hemorrhage similar to those without anticoagulant treatment, suggesting a potential safety advantage. It also identified risk factors for hemorrhage after extraction in patients treated with rivaroxaban [117,118,119]. A study by Ibdah et al. found significant disparities in knowledge and practices among dentists and dental interns from Jordan University of Science and Technology and private clinics, particularly regarding antiplatelet and anticoagulant agents [120,121,122,123,124]. Dental interns with limited clinical experience comprised 23.4% of participants. Knowledge of aspirin usage was high, but newer antiplatelet drugs limited. Only 25% consulted cardiologists [125,126,127,128,129]. The study highlights the need for ongoing education and training in managing anticoagulant therapies in dental patients, emphasizing the importance of workshops, updated curricula, and interdisciplinary collaborations with cardiovascular specialists [62,130,131]. The study by Kwak et al. examined 120 patients receiving Novel Oral Anticoagulants (NOACs) and 153 dental procedures. The study categorized dental procedures into high-risk and low-risk groups based on bleeding risk. Patients not taking NOACs during dental treatment or temporarily taking them were excluded. Dental procedures were performed under local anesthesia, primarily using 2% lidocaine with 1:100,000 epinephrine [47,63,68]. Postoperative bleeding occurred in 9 out of 153 cases, mainly after scaling, extractions, and implant surgeries. The incidence varied, with implant surgeries having a higher proportion. No significant correlation was found [132,133,134,135,136]. The study recommends individualized discontinuation periods for patients on NOAC therapy based on creatinine clearance rates and the type of NOAC used. It also suggests discontinuing NOACs at least 1 day before invasive procedures and fostering collaboration between dental and cardiovascular departments [137,138,139]. The study conducted by Lippert et al. stated that anticoagulation therapy with coumarins is commonly used for patients with cardiac-valve prostheses to reduce thromboembolic risks, despite the bleeding risks associated with such therapy. Invasive dental procedures can be challenging for these patients, necessitating proper information and prophylactic measures for both patients and dentists. Coumarins inhibit vitamin K-dependent enzymes, requiring frequent blood tests to monitor the coagulation system. A study at Rigshospitalet involving 346 patients with cardiac-valve prostheses, primarily treated with phenprocoumon, found that most patients believed their dentists were aware of their anticoagulation treatment. The study suggests that anticoagulation can continue during dental procedures with a temporary INR target of 3.0 or 4.0, supplemented by local antifibrinolytic therapy for additional protection [49]. The study of López Villarreal et al. presented here delves into the investigation of various medicinal plant extracts for their potential biological activities relevant to dental health. In the quest for alternative treatments, the researchers explored the antimicrobial, anticoagulant, antioxidant, cytotoxic, and anti-inflammatory properties of extracts from five different plant species. The research began with the acquisition of dried plant materials from a local store in Monterrey, Mexico. These materials underwent extraction using ethanol, followed by filtration and evaporation to obtain the extracts for further analysis [140,141,142]. Antimicrobial assays revealed that all five extracts exhibited activity against *Streptococcus mutans*, a key bacterium involved in dental caries, with L. graveolens demonstrating the most significant antimicrobial activity. Similarly, the extracts showed varying degrees of activity against *Streptococcus sobrinus*. Further investigations into the anticoagulant activity of the extracts revealed potential effects on prothrombin time (PT) and activated partial thromboplastin time (APTT). While some extracts demonstrated significant alterations in coagulation times, others did not exhibit substantial effects compared to the control [143,144,145]. The study evaluated antioxidant activity of extracts using DPPH radical scavenging, revealing varying potency. Cytotoxicity assays showed concentration-dependent effects on human gingival fibroblasts, emphasizing dose-dependent considerations [146,147,148]. The study found that some plant extracts can modulate inflammation by altering pro-inflammatory and anti-inflammatory cytokines, suggesting their potential as bioactive compounds for developing alternative treatments for dental diseases, requiring further preclinical and clinical trials [55,149,150]. This study of Rubino et al. aimed to assess the frequency and severity of postoperative bleeding events associated with antiplatelet and anticoagulant medication use in patients undergoing common periodontal procedures. The hypothesis was that bleeding frequency would be low regardless of medication use, combination, procedure type, or medication continuation. A retrospective study was conducted using data from patients who underwent periodontal procedures while taking antiplatelet medications, warfarin, or DOACs between 2011 and 2017 [61,151,152]. The study found low bleeding frequency in patients taking antiplatelets, warfarin, or DOACs, and infrequent discontinuation of medications before procedures. Postoperative bleeding events were rare, suggesting current protocols are effective and safe. Further research is needed [153,154,155]. Sammartino et al.’s study investigates the use of platelet-rich fibrin (PRF) as a hemostatic and healing biomaterial during dental extractions in heart surgery patients. Despite concerns about disrupting anticoagulant therapy, the recent literature suggests that continuous therapy may not increase bleeding complications [52,156,157,158]. The study introduces PRF, an autologous platelet concentrate, to prevent postoperative bleeding in heart surgery patients on anticoagulant therapy. The patients were given PRF, prepared from their blood, and monitored for bleeding complications postoperatively [159,160,161]. The study found that PRF, used during dental extractions in heart surgery patients under anticoagulant therapy, effectively prevented postoperative bleeding. It showed good hemostatic properties and facilitated tissue healing without significant adverse events. This suggests PRF could be a valuable adjunct in managing postoperative bleeding in dental procedures [70,162,163]. The rise in chronic medical patients on anticoagulants and antiplatelet medications makes managing oral surgeries, particularly dental extractions, challenging due to increased bleeding tendencies. To address bleeding risks, local antifibrinolytic therapy combined with hemostatic agents is recommended [164,165,166]. Tranexamic acid, used post-surgery, can enhance oral surgical procedures without altering anticoagulant therapy. Oxidized cellulose and fibrin glue, along with tranexamic acid rinses, protect extraction sockets and control bleeding [167,168,169]. Scarano et al.’s study found that CaS application in extraction sockets led to minimal bleeding and normal wound healing, with superior hemostatic properties and no interference with wound healing [53,170,171,172]. The study underscores the importance of maintaining anticoagulant therapy during dental extractions, provided the INR is within therapeutic levels. CaS emerges as a promising hemostatic agent, offering effective bleeding control and facilitating uneventful wound healing [173,174,175]. A study assessed postoperative bleeding in patients undergoing dental extractions while on anticoagulant therapy. Patients were divided into three groups: those using gauze pads, fibrin sponges, or dry gauze compression [54,176,177]. Postoperative bleeding in dental extractions occurs in 4.8% of cases, with advanced periodontal disease being the main risk factor. Three local hemostatic methods are effective in preventing bleeding, and dry gauze compression alone is effective [178,179,180]. Gómez-Moreno et al.’s study assessed the safety and efficacy of dental implant surgery in patients receiving dabigatran therapy, analyzing patient records and clinical data to evaluate bleeding complications, implant success rates, and adverse events [181,182,183]. The study found that dental implant surgery with dabigatran was generally safe and effective, with acceptable bleeding complications. In the Gómez-Moreno et al. study, the researchers investigated the safety and feasibility of dental implant surgery in individuals receiving oral rivaroxaban, an anticoagulant medication [184]. The primary objective of the study was to assess the outcomes of dental implant placement in patients undergoing treatment with rivaroxaban [56,185,186]. The authors conducted a retrospective observational study on dental implant surgery using oral rivaroxaban, finding it generally safe and well-tolerated [187,188,189]. In their prospective study, Pereira et al. aimed to investigate the outcomes of tooth extractions in individuals receiving oral anticoagulant therapy. The study provides insights into the safety and efficacy of dental extractions in this patient population. The primary objective of the study was to assess the incidence of bleeding complications associated with tooth extractions in patients on oral anticoagulants [190,191,192,193]. Their findings likely indicated that tooth extractions in patients receiving oral anticoagulants were generally safe and well-tolerated, with a low incidence of bleeding complications. The study suggested that tooth extractions can be performed safely in patients on oral anticoagulants with appropriate management strategies. However, individual patient factors and the specific anticoagulant regimen should be considered when planning dental procedures to minimize the risk of bleeding complications [51]. Further research, including larger prospective studies, may be beneficial to confirm these findings and provide additional evidence-based recommendations for clinical practice [194,195,196]. In their retrospective observational study, Kim et al. aimed to evaluate the safety and efficacy of DOACs in dental practice. The study provides insights into the management of patients on DOAC therapy undergoing dental procedures. The primary objective of the study was to assess the incidence of bleeding complications and thromboembolic events in patients receiving DOACs undergoing dental procedures [64,197,198,199,200,201,202,203]. The authors’ findings indicated that dental procedures in patients on DOAC therapy were generally safe, with a low incidence of bleeding complications and thromboembolic events. In their clinical study, Karslı et al. aimed to evaluate and compare the bleeding outcomes of dental extractions in patients receiving warfarin versus heparin therapy. The study contributes to the understanding of bleeding risks associated with anticoagulant medications in the context of dental procedures. The primary objective of the study was to assess the incidence and severity of bleeding complications following dental extractions in patients treated with warfarin or heparin [50,204,205]. Through a clinical investigation, the authors examined outcomes such as bleeding duration, volume of blood loss, and the need for additional interventions to control bleeding. Their findings likely indicated that both warfarin and heparin therapy were associated with increased bleeding risk during dental extractions compared to patients not on anticoagulant therapy. However, the severity and duration of bleeding may have varied between the two anticoagulants, with warfarin potentially posing a higher risk compared to heparin [206,207,208,209,210,211,212,213,214,215,216,217,218,219,220,221,222,223,224,225]. Clinicians should consider the type of anticoagulant, its pharmacokinetic properties, and the patient’s overall bleeding risk profile when planning and executing dental procedures to minimize the risk of bleeding complications [226,227,228,229,230,231]. Martinez-Moreno et al. conducted a retrospective study on bleeding complications in patients receiving anticoagulant and/or antiplatelet therapy during dental procedures, providing valuable insights into managing these medications in dental settings [232,233,234]. Through a retrospective analysis, the authors likely examined patient records to gather data on bleeding events, interventions performed to control bleeding, and any adverse outcomes associated with the bleeding episodes. Their findings likely indicated that bleeding complications were relatively common in patients undergoing dental procedures while on anticoagulant and/or antiplatelet therapy. The study likely provided valuable information regarding the types of bleeding events encountered, the effectiveness of hemostatic interventions employed by dental practitioners, and the overall safety of dental procedures in this patient population [235,236,237]. The study likely underscored the importance of careful patient assessment and risk stratification before performing dental interventions in individuals receiving anticoagulant and antiplatelet medications. Dental practitioners should be aware of the potential for bleeding complications and be prepared to manage such events promptly and effectively to ensure patient safety. In conclusion, the findings of this retrospective study likely highlighted the need for clear guidelines and protocols for managing anticoagulated and antiplatelet-treated patients in the dental office [238,239,240,241,242,243,244]. By understanding the risk factors associated with bleeding complications and implementing appropriate preventive measures and management strategies, dental practitioners can optimize the safety and success of dental procedures in this patient population. Further research, including prospective studies and clinical trials, may help validate these findings and refine clinical guidelines for managing anticoagulated and antiplatelet-treated patients in the dental setting [245,246,247]. In their study, Nakamura et al. aimed to investigate post-extraction bleeding episodes in patients receiving antithrombotic therapy. This research contributes to understanding the risk of bleeding complications in individuals undergoing dental extractions while on antithrombotic medication [69,248,249]. The study analyzed post-extraction bleeding events in patients on antithrombotic therapy, using data from the Longevity Improvement and Fair Evidence Study, providing insights into frequency, severity, and management of such bleeding [250,251,252]. The study aimed to identify bleeding complications risk factors and evaluate hemostatic interventions’ effectiveness, emphasizing patient assessment and risk stratification before dental extractions, urging practitioners to promptly manage post-extraction bleeding [253,254,255,256,257,258,259,260,261,262,263,264,265,266,267,268,269]. The study suggests that understanding post-extraction bleeding risk factors and implementing preventive measures can improve patient management of antithrombotic therapy in dental extractions, with further research potentially validating these findings [270,271,272]. In their study, Rocha et al. investigated the impact of perioperative bleeding on postoperative hemorrhage in patients undergoing oral surgery while on antithrombotic therapy. This research sheds light on the role of perioperative bleeding as a potential risk factor for postoperative hemorrhage in this patient population [58,273,274,275,276,277,278,279]. The main aim of the study was likely to evaluate the association between perioperative bleeding during oral surgery and the occurrence of postoperative hemorrhage in patients receiving antithrombotic therapy [280,281,282]. The authors conducted a retrospective analysis on postoperative hemorrhage and perioperative bleeding in oral surgery patients, identifying risk factors and guiding clinical decision-making and patient management strategies [283,284,285]. The study highlighted the need for careful preoperative assessment and risk stratification to minimize the risk of postoperative hemorrhage in patients on antithrombotic therapy undergoing oral surgery. Additionally, it may have emphasized the importance of implementing appropriate hemostatic measures and preventive strategies during the perioperative period to mitigate the risk of bleeding complications [286,287,288]. In their cohort study Rocha et al. aimed to compare bleeding outcomes in individuals undergoing oral surgery who were on anticoagulant therapy versus those who were not. This research provides valuable insights into the impact of anticoagulant therapy on bleeding complications following oral surgical procedures [59]. The primary objective of the study was likely to assess and compare bleeding outcomes, including intraoperative and postoperative bleeding, in patients undergoing oral surgery while on anticoagulant therapy and in a non-anticoagulated control group. The authors likely aimed to investigate whether anticoagulant therapy influenced the incidence and severity of bleeding complications during and after oral surgical procedures [55,289,290]. The study design probably involved prospectively enrolling two groups of patients: one receiving anticoagulant therapy and the other not receiving anticoagulation. The researchers likely collected data on various bleeding parameters, such as intraoperative bleeding volume, postoperative bleeding events, and the need for interventions to control bleeding. Statistical analyses were likely performed to compare outcomes between the two groups. The findings of the study likely provided valuable information on the risk of bleeding complications associated with anticoagulant therapy in the context of oral surgery [291,292,293]. By comparing outcomes between anticoagulated and non-anticoagulated patients, the study likely aimed to elucidate the specific impact of anticoagulation on bleeding outcomes and identify potential risk factors for increased bleeding in this patient population. The study likely highlighted the importance of preoperative assessment and risk stratification to identify patients at higher risk of bleeding complications due to anticoagulant therapy. Additionally, the results may have implications for perioperative management strategies, such as the optimization of hemostatic techniques and the selection of appropriate anticoagulant regimens to minimize bleeding risk while ensuring therapeutic efficacy [294,295,296]. In conclusion, the findings of this cohort study likely contributed to our understanding of the bleeding risks associated with anticoagulant therapy in patients undergoing oral surgery. By elucidating the factors contributing to bleeding complications in this population, the study may have informed clinical practice and guided the development of evidence-based strategies for optimizing surgical outcomes in patients on anticoagulant therapy. Further research in this area may be warranted to validate these findings and refine perioperative management protocols for patients requiring oral surgery while on anticoagulant therapy [297,298,299,300,301,302,303]. Yoshikawa et al. conducted a prospective observational study comparing the safety of tooth extraction in patients on DOACs versus warfarin. The study aimed to assess bleeding complications post-extraction in these two groups, given the rising use of DOACs. Patients were prospectively enrolled into either the DOAC or warfarin group, and data on intraoperative and postoperative bleeding, hemostatic interventions, and adverse events were collected. Statistical analyses compared outcomes between the groups. The findings provided evidence on the relative safety of tooth extractions in patients on DOACs compared to those on warfarin, informing clinical practices and guiding anticoagulant management strategies for dental procedures [60]. In their article, Shah et al. present a case report and discuss the dental management considerations for a patient with a subcutaneous implantable cardioverter defibrillator (S-ICD) who was also undergoing warfarin treatment. This case study addresses a unique clinical scenario involving the coexistence of an S-ICD device and anticoagulant therapy, highlighting the importance of interdisciplinary collaboration and careful perioperative management in dental practice [55]. The primary objective of the article was likely to describe the dental management approach taken for a patient with an S-ICD device and concomitant warfarin therapy, focusing on the considerations, challenges, and precautions involved in providing dental care to such patients. Given the potential risks associated with dental procedures in patients on anticoagulant therapy and those with implantable cardiac devices, the case report likely aimed to provide insights into the safe and effective management of dental treatment in this specific patient population [304,305,306]. The case likely involved a comprehensive assessment of the patient’s medical history, including the indication for S-ICD placement and the rationale for warfarin therapy. Dental treatment planning likely required close coordination between the dental and cardiology teams to minimize the risk of adverse events, including bleeding and cardiac complications. Preoperative evaluation likely included assessing the patient’s INR levels and evaluating the need for temporary discontinuation or modification of anticoagulant therapy to reduce bleeding risk during dental procedures [307,308,309]. The authors likely discussed the specific challenges and considerations associated with dental treatment in patients with S-ICD devices, emphasizing the importance of avoiding electromagnetic interference and minimizing the risk of infection at the implant site. They may have also addressed strategies for managing potential complications such as bleeding and hematoma formation during and after dental procedures, including the use of local hemostatic measures and postoperative monitoring protocols. Overall, the article likely provided valuable insights into the interdisciplinary approach to managing dental care for patients with S-ICD devices and concurrent warfarin therapy [310,311,312]. By presenting a detailed case report and discussing the relevant clinical considerations, the authors aimed to enhance the understanding of dental practitioners regarding the safe and effective management of patients with complex medical conditions requiring dental treatment. The case study likely underscored the importance of individualized treatment planning, close communication between healthcare providers, and adherence to evidence-based guidelines to optimize patient outcomes and minimize the risk of complications in this unique patient population [313,314,315]. Ueda et al. investigated factors contributing to postoperative bleeding following dental extractions in older adults on anticoagulation therapy. The study aimed to identify risk factors to improve clinical management and outcomes. Likely involving a retrospective analysis, the study collected data on patient demographics, medical history, anticoagulant regimen, INR levels, type of extraction, systemic diseases, and other variables. Statistical analyses identified significant predictors of post-extraction bleeding. The findings contributed to understanding bleeding risks in older adults on anticoagulants, informing clinical guidelines and improving perioperative care and patient outcomes [70]. Puia et al. conducted a randomized clinical trial to compare the efficacy of three local hemostatic agents for dental extractions in patients on chronic anticoagulant therapy. They aimed to identify the most effective agent for controlling bleeding and minimizing complications. Standardized protocols and data on patient demographics, medical history, and adverse events were likely used. Statistical analyses compared outcomes between groups. The findings likely guided clinical practice by identifying the most appropriate hemostatic agent, improving patient outcomes, and reducing bleeding risks during dental extractions. The trial provided valuable evidence for managing bleeding in anticoagulated patients [63]. Local hemostatic agents like Histoacryl glue and tranexamic acid mouthwash effectively control bleeding during dental procedures in anticoagulated patients. DOACs, like dabigatran and rivaroxaban, may be a safe alternative. Improved interdisciplinary education and collaboration among healthcare professionals are needed to improve anticoagulant and antiplatelet drug practices. Medicinal plant extracts have potential for alternative treatments [316].

The analyzed studies present several limitations that must be recognized for a correct interpretation of the results. One major limitation is that the review only included articles written in English. This choice may introduce a reporting bias, potentially excluding relevant studies published in other languages that could offer additional evidence or different perspectives. Additionally, many of the studies had small sample sizes, which may limit the generalizability of the findings. Other common limitations include variability in treatment protocols and lack of long-term follow-up to evaluate late complications. Finally, the lack of direct comparative studies between different hemostatic agents and anticoagulant drugs makes it difficult to establish definitive guidelines. Future research should aim to overcome these limitations through larger, multicenter, and inclusive studies of articles in different languages [317].

**Figure 4:**
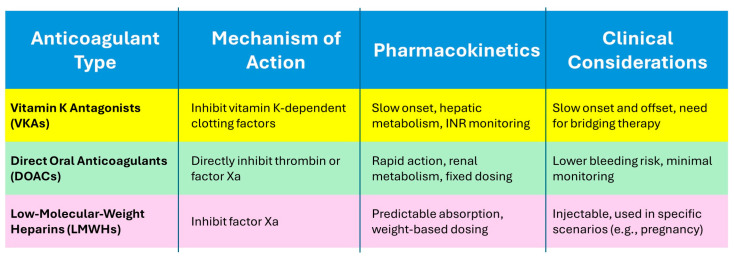
A comparative table that outlines the key characteristics of different anticoagulants relevant to dental practice. Key Points: the mechanism of action describes how each type of anticoagulant affects clotting factors; the pharmacokinetics highlights the absorption, distribution, metabolism, and excretion characteristics; the clinical considerations are practical implications for dental practitioners, such as bleeding risk, monitoring requirements, and specific usage scenarios.

A table format can condense essential information into a straightforward reference, enabling quick understanding and informed decision-making for dental practitioners managing patients on anticoagulant therapy (Figure 4).

## 5. Conclusions

The research we analyzed has made significant progress in understanding and managing dental procedures for patients on anticoagulant or antiplatelet therapy. These studies provide crucial insights that enhance the care of this vulnerable population. Hemostatic agents such as Histoacryl glue, tranexamic acid mouthwash, and fibrin sponges have proven effective in controlling post-extraction bleeding. Researchers showed that tranexamic acid significantly reduced bleeding in anticoagulated patients undergoing oral surgery, and they demonstrated the efficacy of various hemostatic methods. These findings highlight the importance of incorporating effective hemostatic agents into clinical practice to manage bleeding risks. Several studies have investigated the safety and outcomes of dental procedures in patients on anticoagulant therapy. There is the necessity of developing clinical protocols that minimize bleeding risks without the need to interrupt anticoagulant therapy, ensuring both safety and continuity of care. The studies identified several factors influencing bleeding risk, including the type of anticoagulant medication, patient comorbidities, and specific procedure characteristics. There is significant variation in the knowledge and awareness of dentists regarding anticoagulant and antiplatelet therapy. Managing anticoagulated patients often requires close collaboration between dental professionals and other healthcare providers, such as cardiologists and primary care physicians. Such interdisciplinary collaboration is vital to optimize patient outcomes and ensure safety during dental procedures. Understanding individual risk factors, such as the specific type of anticoagulant used and patient comorbidities, allows for the personalization of treatment plans and interventions. Personalized approaches help minimize bleeding risks and improve clinical outcomes. This tailored care approach is essential for effectively managing the complexities of dental care in anticoagulated patients. In summary, the research underscores the transformative impact of evidence-based protocols, comprehensive risk assessment strategies, and interdisciplinary collaborations on dental practice. By adopting these approaches, dental professionals can effectively manage the complexities of providing care to anticoagulated patients, ensuring optimal outcomes and enhancing patient well-being.

## Figures and Tables

**Table 1:** Indicators for database searches.

**Articles Screening Strategy**	KEYWORDS: anticoagulant; dentistry; oral surgery
	Boolean Indicators: “anticoagulant” AND “dentistry”; “anticoagulant” AND “oral surgery”
	Timespan: 1 January 1989, to 7 March 2024
	Electronic databases: PubMed; Scopus; Web of Science.

**Table 2:** A descriptive item selection summary.

Authors	Year	Aim of the Study	Materials	Results
Borea et al. [8]	1993	To investigate the use of tranexamic acid mouthwash in anticoagulant-treated patients undergoing oral surgery	Clinical study evaluating the efficacy of tranexamic acid mouthwash in reducing bleeding after oral surgery in anticoagulant-treated patients	Tranexamic acid mouthwash effectively reduced bleeding after oral surgery in anticoagulant-treated patients
Lippert, S. et al. [49]	1994	To explore the perspectives of cardiac-valve prosthesis patients and their dentists on anticoagulation therapy	Survey study investigating the views of patients with cardiac-valve prostheses and their dentists regarding anticoagulation therapy	Patients with cardiac-valve prostheses and their dentists expressed concerns and uncertainties regarding anticoagulation therapy
Al-Belasy et al. [3]	2003	To evaluate the hemostatic effect of N-Butyl-2-Cyanoacrylate (Histoacryl) glue in warfarin-treated patients undergoing oral surgery	Prospective study comparing the hemostatic efficacy of N-Butyl-2-Cyanoacrylate glue in patients on warfarin undergoing oral surgery	N-Butyl-2-Cyanoacrylate glue is a good alternative hemostatic agent in this population.
Karslı, et al. [50]	2011	To compare the effects of Warfarin and Heparin on bleeding after dental extraction.	Clinical study comparing bleeding outcomes following dental extraction in patients receiving Warfarin or Heparin.	Warfarin and Heparin showed differences in bleeding severity.
Pereira et al. [51]	2011	To examine the outcomes of tooth extraction in patients on oral anticoagulants.	Prospective study involving tooth extraction procedures in patients receiving oral anticoagulants. Evaluation of bleeding complications and associated risk factors.	Tooth extraction in patients on oral anticoagulants showed manageable bleeding complications.
Sammartino et al. [52]	2011	To evaluate the use of leukocyte- and platelet-rich fibrin (L-PRF) in preventing hemorrhagic complications after dental extractions	Clinical study assessing the efficacy of L-PRF in preventing post-extraction bleeding in patients undergoing open-heart surgery and receiving anticoagulant therapy	L-PRF reduced the risk of hemorrhagic complications after dental extractions.
Scarano. et al. [53]	2014	To assess hemostasis control in dental extractions in patients on oral anticoagulant therapy: An approach with calcium sulfate (CaS)	Clinical study investigating the efficacy of CaS in controlling post-extraction bleeding in patients receiving oral anticoagulant therapy	CaS demonstrated effectiveness in controlling post-extraction bleeding.
Soares et al. [54]	2015	To evaluate postoperative hemostatic efficacy of gauze soaked in tranexamic acid, fibrin sponge, and dry gauze compression after dental extractions in anticoagulated patients with cardiovascular disease: A Prospective, Randomized Study	Prospective, randomized study comparing the hemostatic efficacy of gauze soaked in tranexamic acid, fibrin sponge, and dry gauze compression after dental extractions in anticoagulated patients with cardiovascular disease	Gauze soaked in tranexamic acid, fibrin sponge, and dry gauze compression all demonstrated effective postoperative hemostasis.
Shah et al. [55]	2015	To describe the dental management of a patient fitted with a subcutaneous implantable cardioverter defibrillator device and concomitant Warfarin treatment.	Case report describing the dental management approach for a patient with a subcutaneous implantable cardioverter defibrillator device and concurrent Warfarin therapy.	The dental management strategy effectively addressed the treatment needs of a patient with a subcutaneous implantable cardioverter defibrillator device and concomitant Warfarin therapy.
Gómez-Moreno et al. [56]	2016	To assess dental implant surgery outcomes in patients receiving the anticoagulant Oral Rivaroxaban.	Investigation of dental implant surgery outcomes in patients undergoing treatment with Oral Rivaroxaban. Assessment of bleeding complications, implant success rates, and peri-implant parameters.	Dental implant surgery in patients treated with Oral Rivaroxaban demonstrated favorable outcomes with low incidence of bleeding complications.
Gómez-Moreno et al. [57]	2018	To evaluate dental implant surgery outcomes in patients treated with Dabigatran.	Prospective study involving patients undergoing dental implant surgery while on Dabigatran. Evaluation of bleeding complications, implant success rates, and peri-implant parameters.	Dental implant surgery in patients on Dabigatran showed low incidence of bleeding complications.
Rocha et al. [58]	2018	To evaluate perioperative bleeding as a risk factor for postoperative hemorrhage in oral surgery patients.	Investigation of perioperative bleeding as a significant risk factor for postoperative hemorrhage in oral surgery patients.	Perioperative bleeding was identified as a significant risk factor for postoperative hemorrhage in oral surgery patients.
Rocha et al. [59]	2019	To assess bleeding in oral surgery patients on anticoagulant therapy compared to a non-anticoagulated group.	Cohort study comparing bleeding outcomes in oral surgery patients on anticoagulant therapy versus a non-anticoagulated group.	Oral surgery patients on anticoagulant therapy experienced higher rates of bleeding compared to a non-anticoagulated group.
Yoshikawa et al. [60]	2019	To evaluate the safety of tooth extraction in patients receiving direct oral anticoagulant treatment compared to Warfarin.	Prospective observation study comparing the safety of tooth extraction in patients receiving direct oral anticoagulants versus Warfarin.	Tooth extraction in patients receiving direct oral anticoagulants was found to be as safe as in those receiving Warfarin.
Kwak et al. [47]	2019	To investigate bleeding related to dental treatment in patients taking NOACs.	Retrospective study assessing bleeding complications following dental treatment in patients prescribed NOACs.	Dental procedures can be safely performed in patients taking NOACs with appropriate precautions.
Rubino et al. [61]	2019	To investigate postoperative bleeding associated with antiplatelet and anticoagulant drugs.	Retrospective study analyzing postoperative bleeding rates in patients undergoing dental procedures while on antiplatelet or anticoagulant therapy.	Dental procedures in patients on antiplatelet or anticoagulant therapy were associated with a low incidence of postoperative bleeding.
Ibdah et al. [62]	2020	To assess the knowledge and perception of antiplatelet and anticoagulant agents among dentists in Northern Jordan.	Survey study evaluating the understanding and attitudes of dentists in Northern Jordan regarding antiplatelet and anticoagulant therapy.	Dentists in Northern Jordan demonstrated varied knowledge and perceptions regarding antiplatelet and anticoagulant agents.
Puia et al. [63]	2020	To compare three local hemostatic agents for dental extractions in patients under chronic anticoagulant therapy.	Randomized clinical trial comparing the efficacy of three local hemostatic agents for controlling bleeding during dental extractions in patients receiving chronic anticoagulant therapy.	The study compared the effectiveness of three local hemostatic agents in controlling bleeding during dental extractions in patients under chronic anticoagulant therapy.
Kim et al. [64]	2021	To investigate the use of direct-acting oral anticoagulants in dental practice.	Retrospective observational study analyzing the impact of direct-acting oral anticoagulants on bleeding complications in dental procedures.	Use of direct-acting oral anticoagulants in dental practice was associated with manageable bleeding complications.
Martínez-Moreno et al. [65]	2021	To assess bleeding complications in anticoagulated and/or antiplatelet-treated patients at the dental office.	Retrospective study examining bleeding events in patients receiving anticoagulation and/or antiplatelet therapy during dental procedures.	Anticoagulated and/or antiplatelet-treated patients experienced bleeding complications during dental procedures.
Hiroshi et al. [66]	2022	To determine the frequency of hemorrhage after tooth extraction in patients treated with direct oral anticoagulants.	Multicenter cross-sectional study assessing the incidence of post-extraction bleeding in patients receiving DOACs.	Post-tooth extraction hemorrhage occurred at a low frequency in patients treated with DOACs.
López et al. [67]	2022	To investigate the biological activities of five selected plants with therapeutic application in dentistry.	Preliminary study evaluating the antimicrobial, anticoagulant, antioxidant, cytotoxic, and anti-inflammatory properties of five plants used in dentistry.	Selected plants exhibited potential therapeutic benefits in various oral health applications.
Puia et al. [68]	2022	To evaluate bleeding complications in relation to the International Normalized Ratio for dental extractions in patients under chronic anticoagulant therapy.	Evaluative study assessing bleeding complications in relation to the International Normalized Ratio during dental extractions in patients receiving chronic anticoagulant therapy.	Bleeding complications during dental extractions were influenced by the International Normalized Ratio in patients under chronic anticoagulant therapy.
Nakamura et al. [69]	2023	To analyze post-extraction bleeding in patients on antithrombotic therapy.	Analysis of post-extraction bleeding in patients receiving antithrombotic therapy using data from a longitudinal study.	Patients on antithrombotic therapy experienced post-extraction bleeding.
Ueda et al. [70]	2023	To identify factors influencing postoperative bleeding after dental extraction in older adult patients on anticoagulation therapy.	Examination of factors contributing to postoperative bleeding following dental extraction in older adult patients receiving anticoagulation therapy.	Postoperative bleeding after dental extraction in older adult patients on anticoagulation therapy was influenced by various factors, highlighting the complexity of managing bleeding risk in this population.

## Data Availability

Not applicable.

## Abbreviations

ADP: adenosine diphosphate; APTT: activated partial thromboplastin time; DPPH: 2,2-diphenyl-1-picrylhydrazyl; CaS: calcium sulfate; COX: cyclooxygenase; DAPT: dual antiplatelet therapy; DOACs: Direct oral anticoagulants; FWS: fetal warfarin syndrome; INR: international normalized ratio; JUST: Jordan University of Science and Technology; LMWHs: Low-molecular-weight heparins; L-PRF: leukocyte- and platelet-rich fibrin; NOACs: Novel Oral Anticoagulants; NSAID: nonsteroidal anti-inflammatory drug; PRF: platelet-rich fibrin; PRISMA: Preferred Reporting Items for Systematic Reviews and Meta-Analyses; PROSPERO: The International Prospective Register of Systematic Reviews; PT: prothrombin time; S-ICD: subcutaneous implantable cardioverter defibrillator; VKAs: Vitamin K antagonists.
